# AI Optimization-Based Heterogeneous Approach for Green Next-Generation Communication Systems

**DOI:** 10.3390/s24154956

**Published:** 2024-07-31

**Authors:** Haitham Khaled, Emad Alkhazraji

**Affiliations:** Department of Electro-Mechanical Systems Engineering Technology, Abu Dhabi Polytechnic, Abu Dhabi P.O. Box 111499, United Arab Emirates; emad.alkhazraji@actvet.gov.ae

**Keywords:** AI optimization, cognitive radio, software-defined radio, green communication

## Abstract

Traditional heterogeneous networks (HetNets) are constrained by their hardware design and configuration. These HetNets have a limited ability to adapt to variations in network dynamics. Software-defined radio technology has the potential to address this adaptability issue. In this paper, we introduce a software-defined radio (SDR)-based long-term evolution licensed assisted access (LTE-LAA) architecture for next-generation communication networks. We show that with proper design and tuning of the proposed architecture, high-level adaptability in HetNets becomes feasible with a higher throughput and lower power consumption. Firstly, maximizing the throughput and minimizing power consumption are formulated as a constrained optimization problem. Then, the obtained solution, alongside a heuristic solution, is compared against the solutions to existing approaches, showing our proposed strategy is drastically superior in terms of both power efficiency and system throughput. This study is then concluded by employing artificial intelligence techniques in multi-objective optimization, namely random forest regression, particle swarm, and genetic algorithms, to balance out the trade-offs between maximizing the throughput and power efficiency and minimizing energy consumption. These investigations demonstrate the potential of employing the proposed LTE-LAA architecture in addressing the requirements of next-generation HetNets in terms of power, throughput, and green scalability.

## 1. Introduction

Driven by the growing popularity of data-hungry applications and devices such as laptops and smartphones, mobile data traffic is anticipated to overpower the capacity of current mobile communication networks in the near future. As per Cisco’s Visual Networking Index (VNI) [1], the number of smartphones is projected to reach 6.1 billion by 2021, up from 2.6 billion in 2016. These smartphones will account for a sevenfold increase in global mobile data traffic, reaching 48 exabytes/month by 2022. This represents 86% of global mobile data traffic by 2022.

This formidable growth in mobile data traffic comes with numerous technical challenges for next-generation communication systems. Among the challenges, spectrum scarcity and high energy consumption are considered two major concerns. The energy consumption figure in the communication sector is projected to reach 1700 terawatt-hours (TWh) by 2030, up from 600 TWh in 2011 [2].

Owing to this exponential data traffic growth over the last five years and the fact that the licensed spectrum is a scarce resource, current mobile network infrastructures are becoming progressively dense and chaotic. The 5G networks are projected to handle more than a sevenfold increase in data traffic by 2021, where rigorous latency and all-time reliability are all mandatory requirements [3]. To accommodate the unprecedented surge in traffic demand, 5G networks rely on several crucial innovations, chiefly the introduction of heterogeneous networks (HetNets) [4].

HetNets have the potential to effectively address major issues related to spectrum scarcity and energy consumption in next-generation networks. In HetNets, a diverse array of base stations (BSs), including femto, pico, and micro BSs, collectively known as small-cell base stations (SCBSs), are strategically deployed, aiming to alleviate the overall power consumption and spectrum requirements. These small-cell BSs vary in terms of their coverage area and transmission power, allowing HetNets to enhance their capacity by employing an intensive frequency reuse approach within contiguous cells, thereby mitigating spectrum scarcity issues. Both network operators and users stand to benefit from this approach. From the users’ perspective, HetNets bring network infrastructure closer, leading to improved link reliability and quality.

However, managing high-density networks like HetNets, which heavily rely on frequency reuse, presents a challenging task due to the tight coordination among neighboring base stations. This coordination manifests in several ways. Firstly, because of the small cell coverage, user mobility causes rapid fluctuations in network load. Therefore, tasks such as resource allocation, cell association, and handovers must be synchronized among all BSs, including contiguous ones, to minimize signal interference, boost network capacity, balance traffic loads [5], and minimize network energy consumption [6,7]. Secondly, the extensive frequency reuse and broadcast nature of wireless communication result in significant interference experienced by users of one BS from surrounding BSs. This interference negatively impacts the usable capacity of HetNets [5].

Software-defined radio (SDR) technology, which can dynamically update the operating parameters, including the frequency bands, can be quite useful in this context [8]. SDR offers an opportunity for HetNets to adapt to changed network conditions and transcends traditional hardware constraints by replacing fixed hardware components with programmable software algorithms, enabling dynamic signal processing functionalities such as modulation, demodulation, and filtering. This flexibility allows SDRs to support multiple communication standards and protocols through software-defined implementations, eliminating the need for separate hardware for each standard and facilitating easy upgrades as standards evolve. SDRs can be remotely configured and managed, making them suitable for deployment in remote or inaccessible locations and reducing the development time and costs associated with hardware redesign. By focusing on software development rather than hardware, SDRs offer a versatile, adaptable, and future-proof solution to a wide range of radio applications, from amateur radio to military communications [8,9]. As a result, SDR has been an active area of research, where studies have introduced numerous useful models for SDR-based networks in the literature.

**Figure 1:**
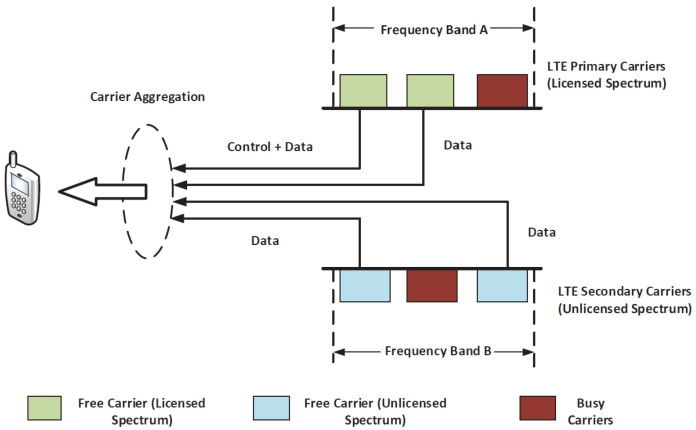
Carrier aggregation in LTE-LAA.

Another promising solution which can greatly enhance the capacity of next-generation networks and deliver a high throughput is long-term evolution licensed assisted access (LTE-LAA). In LTE-LAA, as shown in Figure 1, the control signal and user’s data are carried using primary carriers from the licensed spectrum. However, to boost the data rate, secondary carriers from the unlicensed spectrum are aggregated, using a carrier aggregation technique [10], with the primary carriers to deliver a higher throughput for the user’s equipment [11,12]. The LTE-LAA technique gained approval as part of the 3rd Generation Partnership Project (3GPP) Release 13 [13].

However, current LTE-LAA systems face limitations due to their hardware configurations, restricting them to operating solely within an operator’s predefined licensed bands alongside a fixed unlicensed band, like the 5 GHz frequency range [12]. Consequently, only the subscribers of a specific operator can take advantage of LTE-LAA’s benefits. To extend these benefits to users from various operators, LTE-LAA systems need to support multiple licensed and unlicensed bands, a feat achievable through SDR technology. This advancement would bring advantages to all operators by enhancing revenue and spectrum utilization and benefit users with increased data speeds, improved quality of service, and an overall superior experience quality.

Due to their high potential, both SDR and LTE-LAA systems have been studied reasonably well in the literature. However, almost all of the existing approaches treat SDR and LTE-LAA systems separately. In this work, we present a way to couple SDR with LTE-LAA systems.

In particular, we present a new SDR-based LTE-LAA architecture for HetNets. We present a resource management solution for this architecture where the goal is to decrease energy consumption and increase the overall throughput. To the best of our knowledge, this is the first work to combine the SDR and LTE-LAA concepts together to maximize the throughput and minimize energy consumption in HetNets.

The major contributions of this work can be summarized as follows:

- We introduce a new architecture for HetNets where the SDR and LTE-LAA concepts complement each other.
- We present a resource management scheme for the proposed architecture to maximize network performance with regard to energy consumption and throughput. We formulate this resource management problem as a constrained optimization problem and provide solutions to the optimization problem.
- Considering the time complexity of the optimization model, we introduce a heuristic solution which provides good suboptimum solutions. The proposed solution is then benchmarked against existing solutions.

The remainder of this paper is structured as follows. The next section explores and discusses previous related works and explains how this research covers gaps in previous research studies. In Section 3, we illustrate and explain our system model and its different parameters, including our communication model, traffic model, and energy model. Moreover, we present the heuristic algorithm and its implementation. Section 4 provides a detailed discussion of the obtained results. In Section 5, we utilize AI techniques to perform a multi-objective optimization of the system. Section 6 states the concluding remarks.

## 2. Related Work and Problem Statement

### 2.1. Related Work

Recently, considerable attention has been directed toward HetNets in the realm of research due to their adaptability to the variability in traffic patterns for improving network energy efficiency [14,15], with a wealth of research offering various techniques to enhance their performance. For instance, the authors of [16,17,18] conducted extensive research on optimizing the HetNet backhauling architecture, focusing on minimizing power consumption through the introduction of diverse backhaul solutions. In a similar vein, the authors of [19] delved into utilization of the millimeter-wave spectrum in both backhaul and access networks. Their thorough analysis covered a range of frequency bands, including 73 GHz, 60 GHz, 38 GHz, and 28 GHz, all aimed at enhancing the small cell capacity. Meanwhile, Dehos et al., as explored in [20], assessed the feasibility of millimeter-wave technology for both backhaul and access infrastructures, with a particular emphasis on the V and E bands. In a different approach, the authors of [21] introduced an innovative HetNet backhaul infrastructure which harnessed millimeter-wave and orthogonal frequency division multiple access (OFDMA-PON) in conjunction with software-defined radio, facilitating flexible and cost-effective coverage solutions.

In terms of SDR, ref. [22] presented a comprehensive overview of the SDR concept, elucidating the challenges and potential benefits associated with its adoption in cellular networks. Meanwhile, ref. [23] proposed an SDR-based networking architecture called SoftAir, designed to optimize the allocation of licensed spectrum resources through various traffic-engineering solutions. In a distinct approach, ref. [5] developed a centralized control plane for cellular networks based on SDR technology. Their SoftRAN approach treated all base stations within a specific coverage area as components of a unified virtual BS under a single central controller, aiming to enhance network management efficiency and profitability.

Furthermore, ref. [24] introduced a user-associating scheme which incorporates SDR technology to adapt and distribute the associating algorithm according to traffic load, with the primary objective of reducing overall power consumption and relieving backhaul network burdens. Finally, ref. [25] proposed a mobility-based user-associating scheme suitable for cooperative communication scenarios.

The HetNet concept is highly sensitive to user–cell association rules due to significant disparities in BSs’ coverage areas. Incommensurate cell coverage areas lead to unequal traffic loads in the case of a maximum signal-to-interference-and-noise ratio (SINR) cell association, presuming that the mobile user distribution is relatively uniform. The load difference in a macro cell-only network is limited because the size of almost all cells’ coverage areas is the same. However, the opposite is true in HetNets. This leads to a higher level of complexity with regard to the user association problem in HetNets. Recent studies on user association can be categorized into two groups:

- Group (1) uses techniques based on borrowing channels from underutilized BSs, such as hybrid channel assignment (HCA) in [26], load balancing with selective borrowing (LBSB), and channel borrowing without locking (CBWL) [27].
- Group (2) uses techniques based on transferring traffic to underutilized BSs, such as hierarchical macrocell overlay system cell zooming [28,29] and biasing methods [30].

The subject of traffic load balancing within HetNets has garnered substantial attention in recent research endeavors. Various algorithms have emerged with the aim of achieving effective traffic load distribution in HetNets, thereby optimizing network resources. For instance, in [31], the authors approached the challenge of load balancing as an optimization problem, focused on maximizing efficiency. Furthermore, they introduced a user association algorithm rooted in primal-dual decomposition. In a different vein, ref. [32] saw Aryafar et al. conceptualizing traffic load balancing as a congestion game, where the association decisions act as actions and the network users assume the roles of players.

The authors of [33] offered a user association algorithm which achieves load-balancing in HetNets while utilizing green energy-powered BSs to decrease on-grid energy consumption. In [34], the authors presented simple and efficient user association rules in the form of closed-form formulas which magnify the utility of the network. In [35], the author presented an evolutionary approach which considers both user association and power allocation while balancing loads in HetNets. In [36], flexible cell-breathing techniques were introduced for application within HetNets. These techniques enable the transfer of loads from overloaded base stations to neighboring, lightly loaded BSs. The primary objectives of these techniques are to enhance energy efficiency and achieve load balancing. Extensive surveys have been conducted by the authors of [30,37], encompassing a comprehensive review of available methods for traffic load balancing and BS biasing within HetNets, all aimed at improving energy efficiency and overall performance. Furthermore, the authors of [38,39,40,41] covered a range of various similar aspects within HetNets, including SDR architectures, interference coordination, utilizing SDR in small-cell deployments, SDR energy efficiency, and the application of machine learning techniques for resource management. They provided comprehensive insights into current research trends and challenges in heterogeneous network environments.

The majority of the aforementioned research endeavors have concentrated on employing various technologies such as mmWave [42] bands, FTTB, FTTN, VDSL2, and SDR to alleviate backhaul traffic, reduce costs and latency, expand coverage areas, and enhance energy efficiency. However, it is worth noting that most of these works have relied on wireless network designs which are heavily constrained by preconfigured hardware and constructed upon closed, rigid, and non-adaptable architectures. Such designs typically pose significant challenges in adapting to unpredictable, variable, and continuously growing traffic patterns [43].

Furthermore, due to the inflexible hardware-based design and the scarcity of licensed spectrum resources, the focus of these works has primarily been on maximizing the efficiency of the allocated licensed spectrum rather than optimizing the achievable data rates for users. Moreover, influenced by the closed hardware-centric approach, many of the aforementioned authors have not explored the possibility of using BSs that can be shared by multiple operators, which could potentially reduce operational costs and overall energy consumption.

### 2.2. Problem Statement

Due to its preconfigured and hardware-constrained design, an existing LTE-LAA network only operates in an allocated licensed band along with an unlicensed band [44,45]. Consequently, only the users who have access to this allocated licensed band of the LTE-LAA network can enjoy the benefit of a higher throughput. The full potential of the LTE-LAA system can be further realized if users from multiple operators (i.e., multiple licensed bands) can access this LTE-LAA network. SDR can be an enabling technology in this context. The research question becomes how to couple SDR with the LTE-LAA system and manage resources in this SDR-enabled LTE-LAA network so that users, irrespective of their operators, can benefit from the LTE-LAA network in the form of a higher throughput and lower energy consumption.

In this work, we propose an approach which utilizes SDR technology to introduce open-access or shared, SDR-based BSs which support LTE-LAA and can serve users from different operators. The proposed approach adds flexibility to HetNets, increases the overall data rate for the users, and enhances energy efficiency. Hence, its benefits can be realized by both operators and users.

## 3. Proposed SDR-Based LTE-LAA Architecture and Its Resource Management Scheme

This section presents a description of our SDR-based LTE-LAA system model, which comprises a network model, SDR-based BS communication model, traffic model, and energy model.

### 3.1. Network Model

**Figure 2:**
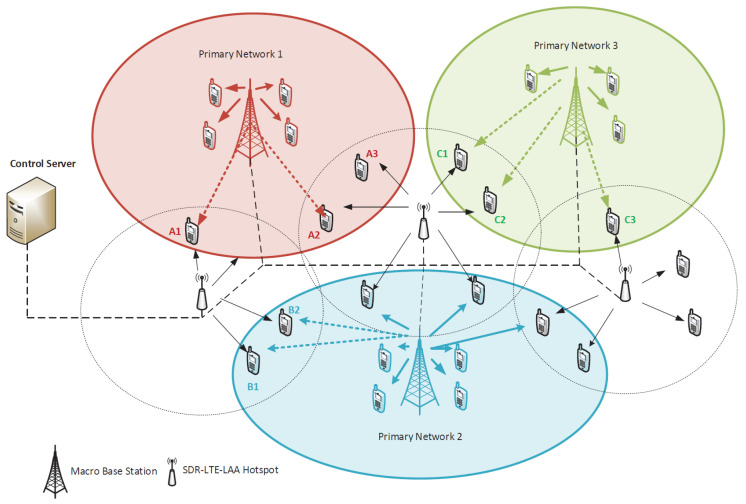
System model.

The architecture of our SDR-based LTE-LAA system is illustrated in Figure 2. The system consists of macro base stations (MBSs), multiple SDR-based small-cell BSs, a control server, and end users. We represent the user set as $J=\{J_{1},J_{2},J_{3},\cdots ,J_{N}\}$, indexed by *j*, and represent the BS set as $I=\{I_{1},I_{2},I_{3}\cdots ,I_{M}\}$, indexed by *i*. Small cell BSs are assumed to support the LTE-LAA technique, where a BS uses the carrier aggregation concept and combines LTE carriers from both the unlicensed and licensed spectra [10]. Furthermore, these SDR-based BSs are distributed in consonance with a spatial Poisson point process (PPP) at a density of $\varepsilon_{0}$ around the MBSs, where $\varepsilon_{0}$ represents the median number of small-cell BSs per unit area [46,47]. Thus, this system model presents a two-tier heterogeneous network where MBS are overlaid with multiple SDR-based small-cell BSs. In our system model, the MBS covers several SCBS and various users. Owing to the temporal and spatial dynamics of the mobility of users, some SCBSs are underutilized, and others are fully loaded with their native users. Hence, the underutilized SCBSs can offer, for a fee, their unused bands to the cognitive, radio-enabled users of the MBSs, accessed via the cognitive radio technique. Using SDR, the cognitive radio technique allows the device to scan various licensed bands to detect a free band or spectrum and send it to the control server. Once complete, the control server controls the user’s association to the optimum base station for a fee. It is worth highlighting that in such a paradigm, if the primary user (i.e., operator customer) becomes available, then secondary users (i.e., not the operator customer) will be requested by the control server to leave the band. Accordingly, this is a win-win situation, where an underutilized BS which belongs to any operator can be utilized to earn more profit by serving secondary users from different operators for a fee.

The SDR-based small-cell BSs scan the spectral environment continuously to find and generate two lists. Firstly, there is $L_{1}$, which is a list of unused or idle bands in both the licensed and unlicensed spectra in their coverage areas. This list is used as a base to establish the carrier aggregation for the LTE-LAA technique. Secondly, there is $L_{2}$, which is a list of the nearby macro-connected users and their utilized channel frequencies. This list represents the potential users to be offloaded to the SDR-based small-cell BSs.

As illustrated in Figure 2, a control server is deployed and connected to all BSs in the area, including the macro BSs. This control server receives periodic updates from the BSs. These updates include $L_{1}$, $L_{2}$, the BSs’ loads, radio element utilization, power consumption, and interference levels. These updates help the server to develop a comprehensive overview of the traffic load and its distribution all over the coverage area and to develop an interference map for this coverage area.

The control server, which now has the knowledge of all the BS states in the entire coverage area, can easily recognize the best user allocation which maximizes the total throughput, minimizes the overall power consumption, and balances the load between BSs. Accordingly, it generates $L_{3}$, which contains the optimum user-BS association list along with the number of user-allocated carriers and notifies the SDR-based BSs to act accordingly. Using $L_{3}$ and the SDR capabilities, the BSs adjust their transmission parameters to perform carrier aggregation and provide LTE-LAA technology to the surrounding users. This aggregation provides users with faster data rates and a more responsive experience.

To further clarify the functionality of the control server, as shown in Figure 2, users A1, A2, A3 (from network 1), B1, B2 (from network 2), C1, C2, and C3 (from network 3) are edge users at their vendor networks. Upon receiving and processing the updates, the control server can recognize the benefits which edge users may achieve if they are offloaded to the nearby SDR-based BSs, particularly if these nearby BSs can supply them with far more radio resource blocks or carriers using LTE-LAA technology. Hence, it generates a new optimum user–BS association list and notifies the SDR-based BSs to adapt accordingly. In peak hours, such a scenario requires substantial procedures and communications between BSs, whereas the control server can easily determine the allocation which balances the load and issue an instruction to the SDR-based BSs to act accordingly.

It is worth noting that offloading the above-mentioned users to nearby BSs allows the macro BSs to use cell zooming techniques [30] to reduce their power consumption and save a considerable amount of power, as the BS is responsible for more than 60% of the overall network power consumption [48].

### 3.2. SDR-Based Base Station Communication Model

The BS transmission power is denoted as $P_{i}$, and the gain of the channel between a BS *i* and a user *j* is denoted as $g_{ij}$. Meanwhile, $\sigma^{2}$ denotes the noise power. Hence, the signal’s SINR from a BS *i* toward a user *j* can be given by

(1) $$\alpha_{ij}=\frac{P_{i}\cdot g_{ij}}{\sum_{v\in I,v\neq i}P_{v}\cdot g_{iv}+\sigma^{2}}$$

In this study, we adjust the modulation and coding scheme (MCS) after evaluating the behavior of the channel based on γ (channel attenuation factor). This approach is known as the maximum throughput channel state [49]. We use this approach to achieve the maximum throughput by adapting the MCS in accordance with the SINR value. This is a suitable approach for streaming and conversational traffic [50].

In a Rayleigh channel, the error probability in a symbol can be calculated as follows [51]:

(2) $$\begin{array}{cc} P_{e} & =\frac{4(\sqrt{m}-1)}{\sqrt{m}}\cdot Q\left(\sqrt{\frac{3\cdot \alpha_{ij}\cdot \gamma^{2}}{\left(m-1\right)}}\right)-{\left[\frac{2(\sqrt{m}-1)}{\sqrt{m}}\cdot Q\left(\sqrt{\frac{3\cdot \alpha_{ij}\cdot \gamma^{2}}{\left(m-1\right)}}\right)\right]}^{2} \end{array}$$

where *m* denotes the modulation order.

The bit error probability $P_{e}^{b}$ is a function of $P_{e}$. Hence, it can be expressed as follows:

(3) $$P_{e}^{b}≅\frac{P_{e}}{log_{2}\left(m\right)}$$

By assuming that the fading is independent from one frame to another and utilizing the maximum likelihood algorithm, the probability of receiving an error-free block can be calculated as follows [51]:

(4) Pefblk=∑q=0QWqPebq(1−Peb)W−q

where *W* denotes the codeword width and *Q* is the number of bits. Hence, the block error probability can be calculated as follows:

(5) $$P_{e}^{blk}=1-P_{ef}^{blk}$$

Accordingly, the max data rate (in bps) between the BS *i* and user *j*, based on the channel condition, can be derived as follows:

(6) $$D_{ij}^{max}=\frac{N_{i}^{c}\cdot N_{symb}\cdot R_{w}\cdot log_{2}\left(m\right)}{T_{fr}}\cdot \left[1-P_{e}^{blk}\right]$$

where $N_{c}^{i}$ is expressed as

(7) $$N_{i}^{c}=N_{l}^{i}+N_{u}^{i}$$

where $N_{i}^{c}$ presents the total number of assignable subcarriers at BS *i*, $N_{l}^{i}$ depicts the number of subcarriers in the licensed band, $N_{u}^{i}$ represents the number of subcarriers in the unlicensed band, $N_{symb}$ is the number of symbols, $R_{w}$ is the code rate of the used MCS, and the frame time duration is denoted by $T_{fr}$.

### 3.3. Traffic Model

In this work, it is assumed that each user’s traffic reaches the BS *i* in line with a Poisson process, with flow lengths of an average value $l_{j}$ and an arrival rate $r_{j}$. Thus, a user *j* generates an average traffic load density in a BS *i* according to the following:

(8) $$\varepsilon_{ij}=\frac{r_{j}\cdot l_{j}\cdot \left(x_{ij}+y_{ij}\right)}{D_{ij}^{max}}$$

where $x_{ij}$ is a binary decision variable which shows if the *j*th user is connected to the *i*th BS or not. Hence, we have

(9) $$x_{ij}=\left\{\begin{array}{cc} 1 & \mathrm{if}\;\mathrm{user}\;j\;\mathrm{is}\;\mathrm{connected}\;\mathrm{to}\;\mathrm{BS}\;i\\ 0 & \mathrm{otherwise} \end{array}\right.$$

In addition, $y_{ij}$ takes on the value of 1 if at least one subcarrier is available at BS *i* and is 0 otherwise. Accordingly, $y_{ij}$ is expressed as follows:

(10) $$y_{ij}=\left\{\begin{array}{cc} 1 & \mathrm{if}\;j\mathrm{th}\;\mathrm{user}\;\mathrm{is}\;\mathrm{connected}\;\mathrm{with}\;\mathrm{BS}\;i\;\mathrm{using}\;\mathrm{unlicensed}\;\mathrm{carrier}\\ 0 & \mathrm{otherwise} \end{array}\right.$$

The average traffic load density for a BS *j* can be calculated by aggregating the average load densities for all users connected to this BS as follows:

(11) $$\varphi_{i}=\sum_{j\in J}\varepsilon_{ij}$$

While the users’ traffic arrival rate $r_{j}$ is in line with the Poisson process (PP), the time of service for each user’s load follows the general distribution. Thus, a BS *i* realizes a sharing queue of the M/G/1 process type. The BS’s user is expected to generate a data load $l_{j}$ with a data rate $D_{ij}$. Additionally, $\varphi_{i}$ needs to be less than 1 to have a stable queue. In this queue, the average time of service (in seconds) for a user *j* is calculated as follows:

(12) $$T_{ij}=\frac{l_{j}}{D_{ij}^{max}\cdot \left(1-\varphi_{i}\right)}$$

The effective data rate (i.e., throughput in bps) for each user can be expressed as follows: [52,53]:

(13) $$D_{ij}^{eff}=D_{ij}^{max}\cdot \left(1-\varphi_{i}\right)$$

where $D_{ij}^{eff}$ is the effective data rate for each user *j* connected to a BS *i*. Thus, the total throughput of a BS *i* is expressed (in bps) as follows:

(14) $$\eta_{i}=\sum_{j\in J}D_{ij}^{eff}$$

### 3.4. Energy Model

The user’s power consumption comprises two types: static and dynamic. The static power $p_{j}^{st}$ is consumed by the user’s smartphone to remain on and running without data exchange, and it is assumed to be constant. The dynamic power $p_{j}^{dy}$ is consumed by the user’s smartphone while communicating with the BS. This power depends on the number of used carriers and the distance of the user *j* from the BS *i*. Hence, the user’s total power consumption (in Watts) can be formulated as follows:

(15) $$p_{j}=p_{j}^{st}+\psi_{j}p_{j}^{dy}$$

where $\psi_{j}$ is a linear coefficient which represents the relationship between the data traffic load and the dynamic power.

In this paper, our main focus is to reduce the dynamic power, which is dependent on the user and BS association. In line with the Friis transmission equation, the user’s transmission power can be calculated (in Watts) as follows:

(16) $$P_{ij}=K\cdot \mu_{ij}\cdot x_{ij}$$

where *K* is a constant and can be expressed by

(17) $$K=\frac{16\pi^{2}\cdot p_{r}}{C^{2}}$$

where the speed of light is C and $p_{r}$ denotes the minimal detectable power which is recognizable by the BS. However, $\mu_{ij}$ is a dynamic term (with units of Hz·m^2^) and depends on the assigned subcarrier frequencies and the measured distance between the user *j* and the BS *i*. Accordingly, $\mu_{ij}$ is formulated as follows:

(18) $$\mu_{ij}=\frac{d_{ij}^{2}}{G_{j}\cdot G_{i}}\cdot \sum_{z=1}^{t}f_{z}^{i}$$

where $d_{ij}$ is the measured distance, $G_{j}$ and $G_{i}$ are the antenna gain for the user and BS, respectively, t represents the total number of assignable subcarriers, and $f_{z}^{i}$ represents the frequency of the assigned subcarrier *z* of a BS *i*.

### 3.5. Resource Management Problem Formulation

The resource management scheme for the SDR-based LTE-LAA system has two main targets. The first one is to boost the aggregated throughput of the users, and the second one is to minimize the power consumption of battery-constrained devices. For the users’ throughput, the small-cell BSs assign more subcarriers to users to maximize their overall throughput. These subcarriers are selected from both the licensed and unlicensed spectra using LTE-LAA technology. For users to reduce power consumption, they need to be offloaded from the macro BS to their nearest small-cell BSs. However, this offloading process can lead to congestion in some BSs, which may result in traffic delays and a decrease in the data rate. Accordingly, a user association technique needs to be used to avoid this situation. The proposed technique is explained in more detail later in the following subsection.

Once the throughput and power consumption figures for the users are calculated using the traffic and energy models, the subsequent step involves associating the users to their optimum small-cell BSs so that the two targets are achieved. This is where the small-cell BS scans the surrounding users and selects the optimum set of nearby users for connecting. This decision will be based on the total throughput the user can have by utilizing the unlicensed spectrum and the total power consumed in this process. This can be expressed as a network utility function as follows:

(19) $$z=\sum_{i\in I}\sum_{j\in J}\left(D_{ij}^{eff}+\beta \cdot \left(1-\frac{P_{ij}}{P_{j_{max}}}\right)\right)$$

where $P_{j_{max}}$ is the user’s maximum affordable transmission power, $P_{ij}$ is the power consumed to connect to the SCBS, and $D_{ij}^{eff}$ is the users’ effective data rate (i.e., throughput). The users’ gain is calculated by the first term, and the users’ power consumption represents the users’ cost and is calculated by the second term. Furthermore, β presents a trade-off coefficient, which is set by the user when choosing the power performance modes in his or her smartphone (i.e., normal, power saving, or ultra power saving modes). Accordingly, β is responsible for adjusting the relation between users’ power consumption and their overall throughput.

In order to maximize the overall network benefit, taking into consideration both the user throughput and power consumption, we formulate an optimization problem where $x_{ji}$ is a binary decision variable which takes on the value 1 if a user *j* is connected to a BS *i* and is 0 otherwise. Hence, the objective function *z* expresses the aggregated benefit and is written as follows:

(20) $$\mathrm{maximize}\;z=\sum_{i\in I}\sum_{j\in J}\left(D_{ij}^{eff}+\beta \cdot \left(1-\frac{P_{ij}}{P_{j_{max}}}\right)\right)$$

subject to

(21) $$\sum_{i=1}^{I}x_{ij}\leq 1,\;\forall j\in J$$

(22) $$\sum_{i=1}^{I}D_{ij}^{eff}\geq D_{th},\;\forall j\in J$$

(23) $$y_{ij}\leq x_{ij},\;\forall i\in I$$

(24) $$\sum_{j=1}^{J}x_{ij}\leq N_{l}^{i},\;\forall i\in I$$

(25) $$\frac{\sum_{j=1}^{J}x_{ij}}{N_{u}^{i}}\leq y_{ij},\;\forall i\in I$$

(26) $$P_{ij}\leq P_{j_{max}},\;\forall i\in I$$

(27) $$\varphi_{i}\leq \varphi_{i_{max}},\;\forall i\in I$$

(28) $$x_{ij}\cdot \alpha_{ij}\geq \alpha_{jmin},\;\forall j\in J$$

(29) $$\sum_{j=1}^{J}D_{ij}^{eff}\leq \eta_{i}\;\forall i\in I$$

Here, the following apply:

- Equation (21) makes sure that each user can be assigned to only one small-cell BS.
- Equation (22) indicates that only users who have data demands over a certain threshold are allowed to be offloaded and become connected to another suitable BS to avoid congestion in the network.
- Equation (23) makes sure that users cannot be associated with BSs which have only carriers in the unlicensed band.
- Equation (24) ensures that the number of users associated with any BS should be equal to or less than the number of licensed carriers. This is to meet the LTE-LAA requirement [54].
- Equation (25) ensures that unlicensed carriers are equally distributed between the connected users.
- Equation (26) denotes that the power required by a user *j* to become connected to a BS *i* should not exceed the maximum transmission power $P_{j_{max}}$ which the user can afford based on its available battery power.
- Equation (27) ensures that each BS’s traffic load is less than a maximum threshold $\varphi_{i_{max}}$ which the BS can afford.
- Equation (28) ensures that the SINR between the user and the assigned BS is the best for better seamless communication.
- Equation (29) makes sure that the total data demands of the SUs do not exceed the total idle capacity of the PBS (η).

We solved this optimization problem using the IBM CPLEX studio to find the optimum solution. The optimization problem in Equation (20) is an integer linear programming (ILP) problem, which is challenging to solve in real time. We prove that the above problem is NP-hard. Accordingly, the real-time application is not practical, especially for mobile networks which cover large areas with a huge number of users and BSs.

**Lemma** **1.** *The optimization problem in Equation (20) can be considered an NP-hard problem.*

**Proof.**  Assume that we have two BSs which are located at the same point, and hence, a user’s traffic load toward any of them is the same. In order to prove that Equation (20) is NP-hard, its congruous decision problem needs to be NP-complete. The decision problem could be written as follows:

(30) $$\sum_{i\in I}\sum_{j\in J}D_{ij}^{max}\cdot \left(1-\frac{r_{j}\cdot l_{j}\cdot \left(x_{ij}+y_{ij}\right)}{D_{ij}^{max}}\right)+\beta \sum_{i\in I}\sum_{j\in J}\left(1-\frac{P_{ij}}{P_{j_{max}}}\right)\leq b$$

where *b* is a positive value. Can we then obtain an appropriate solution where *x* = {$x_{ij}|j\in J,i\in I$} such that the constrains in Equations (21)–(23), (25), (26), (28), and (29) are satisfied?

To prove that Equation (30) is NP-complete, we consider only two small-cell BSs installed at the same location, and they have the same maximum traffic load threshold. In other words, we have

(31) $$\varphi_{i_{max}}=\frac{1}{2}\sum_{j\in J}\varepsilon_{ij}+\epsilon$$

where ϵ is considered a small positive value. Hence, we have

(32) $$\epsilon <<\frac{1}{2}min\left\{\varepsilon_{ij}|j\in J\right\}$$

Moreover, suppose that *b* equals +∞. Consequently, Equation (30) is always satisfied under all values of *x*. Additionally, to meet the constraint in Equation (26), it is required to guarantee the following:

(33) $$\sum_{j\in J}x_{j1}\varepsilon_{j1}=\sum_{j\in J}x_{j2}\varepsilon_{j2}=\frac{1}{2}\sum_{j\in J}\varepsilon_{ij}$$

Thus, a partition problem could be generated from the previous decision problem, where the set of users is divided into two separate sets in order to make the traffic load equal for the two sets. Consequently, this partitioning problem can be reduced to Equation (30). Because the partition problem is considered an NP-complete problem, Equation (20) presents an NP-complete formula. Hence, it is considered an NP-hard problem. To address the problem in real time, in the next section, we propose a heuristic solution. □

### 3.6. Heuristic Solution

This section presents our proposed throughput- and energy-aware (TEA) heuristic solution to increase the gross network’s throughput and reduce the total network power consumption. Heuristic algorithms are essential for addressing non-deterministic polynomial time (NP) problems due to their practicality in finding solutions efficiently, given the exponential complexity of exact solutions. They provide timely results which, though not guaranteed to be optimal, meet acceptable standards for real-world applications. Heuristics are crucial in fields like scheduling and resource allocation, where exact solutions are often impractical. Their adaptability allows them to be tailored to specific problem instances, utilizing iterative techniques to refine solutions incrementally. By balancing computational feasibility with solution quality, heuristics play a vital role in optimizing decision-making processes across various complex scenarios The proposed heuristic is summarized in Algorithm 1, and the relevant procedures are illustrated in Algorithms 2 and 3. The heuristic was designed to select the optimum user–BS association, and it was designed to be flexible and adaptable to the rapidly changing nature of traffic demands. The heuristic’s main idea is to find, iteratively, the user that has the smallest utility value and reassign it to another BS to increase the overall network utility. In the algorithm, user *j*, which is allocated to BS *i*, has a network utility value calculated as follows:

(34) $$z_{ij}=D_{ij}^{eff}+\beta \cdot \left(1-\frac{P_{ij}}{P_{j_{max}}}\right)$$

First, users of the networks are assigned to their vendor macro BS which provides the best SINR. Algorithm 1 follows a systematic approach across multiple lines. Specifically, it iterates over each time slot (line 3), initializes the user–BS assignment matrix Π (line 4), adds all users to the initial group $j^{\prime }$ (line 5), and continues as it processes the user–BS associations. This initial allocation ensures that all users are grouped into an initial set $j^{\prime }$. In this process, it selects a user to minimize the utility function (line 8), adds all BSs to the initial group (line 9), and proceeds with Algorithm 2 (line 10). Depending on the condition, it updates the total utility value $Z_{total}$ (line 13) or moves the user to the final group (line 15). This iterative process continues until all users are appropriately associated with BSs and returns the final optimized user–BS association list (line 19). In every iteration, the proposed algorithm selects the user with the smallest utility value among all users to prioritize those with the least favorable network conditions.

Using Algorithm 2, a new BS is identified to increase the user’s utility value, thereby improving the overall network utility. If no suitable BS is found, then the proposed algorithm ignores that user and starts a new iteration. Algorithm 2 aims to optimize user assignment to a base station (BS). Initially, it identifies the set $V_{j*}$ for a specific user j∗ where the utility $u_{ji}$ exceeds a minimum threshold $u_{min}$ (line 1). The set $V_{j}$ is then sorted in decreasing order (line 2). An initial flag *F* is set to 0 (line 3). The algorithm enters a while loop which continues until $V_{j*}$ is empty (line 4). Within the loop, it identifies the optimal BS *i* (line 5) and removes it from the set $I^{\prime }$ (line 6). The assignment indicator $\Pi_{ji}$ is set to 1 (line 7). It calculates the new objective $Z_{new}$ and the performance $P_{ji}$ (line 8). If $P_{ji}$ is less than a maximum threshold $P_{jmax}$ (line 9), then it checks if $Z_{new}$ is greater than the current $Z_{current}$. If this is true, then it updates the assignment Π (line 11) and sets the flag *F* to 1 (line 12). If $P_{ji}$ is not less than $P_{jmax}$, then it proceeds with Algorithm 3 (line 15). The while loop ends when $V_{j}$ is empty (line 17).
**Algorithm 1** The TEA Algorithm.   1:**Input:** Users j∈J; required data rate $D_{j}$; user–BS distances *d*;   2:**Output:** optimum user–BS association list that maximizes the unlicensed throughput and minimizes the overall power consumption;   3:**for** time slot, t∈1,2,⋯,T **do**   4:      Set initial user–BS assignment matrix Π;   5:      add all users to initial group $J^{\prime }$;   6:      **while** $J^{\prime }\neq \emptyset$ **do**   7:            Set *F* = 0;   8:            Find $j^{*}$ from $j^{*}=argmin_{j\in J}\left(u_{j}\right)$;   9:            Add all BSs to initial group $I^{\prime }$; 10:            Go to process Algorithm 2; 11:            **if** *F* == 1 **then** 12:                  Let Δ == $\hat{\Delta }$; 13:                  update $Z_{total}$; overall utility value; 14:            **else** 15:                  Move user to final group *x*; 16:            **end if** 17:      **end while** 18:**end for** 19:**return** x;

Algorithm 3 processes users in a BS based on certain criteria. Initially, it identifies the users in the BS (line 1) and sorts them in ascending order (line 2). It then iterates through each user (line 3). For each user, it checks if the effective delay $D_{iy}^{eff}$ exceeds a threshold $D_{th}$ (line 4). If the condition is met, then it proceeds to Algorithm 2 (line 5). Within this process, if a flag *F* equals 1 (line 6), then a variable Δ is updated to $\Delta_{i}$ (line 7), and the algorithm exits (line 8). If the flag condition is not met, then the algorithm continues to the next user (lines 9–12).
**Algorithm 2** TEA Algorithm 2.   1:Find $V_{j*}$ for user j∗ where $V_{j*}$ = $i|u_{j*i}>u_{min},i\in I^{\prime }$;   2:Sort $V_{j*}$ in decreasing order;   3:set *F* == 0;   4:**while**$V_{j*}\neq \emptyset$**do**   5:      Find optimum BS by $i=argmax_{i\in V}\left(u_{j*i}\right)$;   6:      Remove *i* from $I^{\prime }$;   7:      Set $\Pi_{j*i}$ = 1;   8:      Calculate current $Z_{new}$ and $P_{ji}$ based on new assignment;   9:      **if** $P_{ji}$ <$P_{J_{max}}$ **then** 10:            **if** $Z_{new}$ >$Z_{current}$ **then** 11:                 Set Π == $\hat{\Pi }$; 12:                 Set *F* == 1; 13:            **end if** 14:      **else** 15:            Go to process with Algorithm 3; 16:      **end if** 17:**end while**

**Algorithm 3** TEA Algorithm 3.
   1:Find users in BS *i* using $J_{i}$ = {$j|x_{j*i}=1\&j\in J^{\prime }$};   2:Sort users in an ascending order;   3:**for** *Y* = 1: $|J_{i}|$ **do**   4:      **if** $D_{iy}^{eff}$ >$D_{th}$ **then**   5:            Go to process with Algorithm 2;   6:            **if** *F* == 1 **then**   7:                  Δ == $\hat{\Delta }$;   8:                  exit;   9:            **end if** 10:      **end if** 11:      *Y* = *Y* + 1 12:
**end for**



## 4. Simulation Results and Discussion

This section presents and explains the obtained results along with a comprehensive discussion about our findings. Furthermore, we present a comparison between our proposed strategy with the best SINR user association strategy [55], termed B-SINR, and the load-aware user association strategy, termed LAUA in our paper [56]. The results of this part of the study were obtained by utilizing both IBM ILOG CPLEX Optimization Studio and MATLAB R 2016b. Our simulations were executed on a high-performance PC equipped with an Intel Core i7 processor clocked at 3.8 GHz with 32 GB of RAM. The time it took to run the proposed algorithms to find the optimal solution was 95 s. This was a relatively acceptable delay which allowed the proposed algorithms to be utilized in real-life networks. The simulations were rigorously iterated 50 times to ensure robustness, with the subsequent average values computed and consolidated to form the foundation of our numerical results.

The best SINR strategy associates users with the base station which has the highest SINR. However, the load-aware user association strategy associates users with BSs based on the BSs’ traffic loads. Leveraging SDR, the cognitive radio technique empowers devices to dynamically explore licensed bands, identifying unutilized spectra which can be repurposed. These valuable data are then relayed to a central control server for further orchestration. Once received, and using the proposed algorithms, the control server orchestrates user association to the most optimal base station, with compensation involved. Notably, in this model, if the primary user (i.e., operator customer) resumes activity, then the control server proactively requests secondary users to gracefully exit the allocated band. This strategy fosters a mutually beneficial environment wherein dormant base stations, irrespective of ownership, can unlock additional value by catering to secondary users from diverse operators.

**Figure 3:**
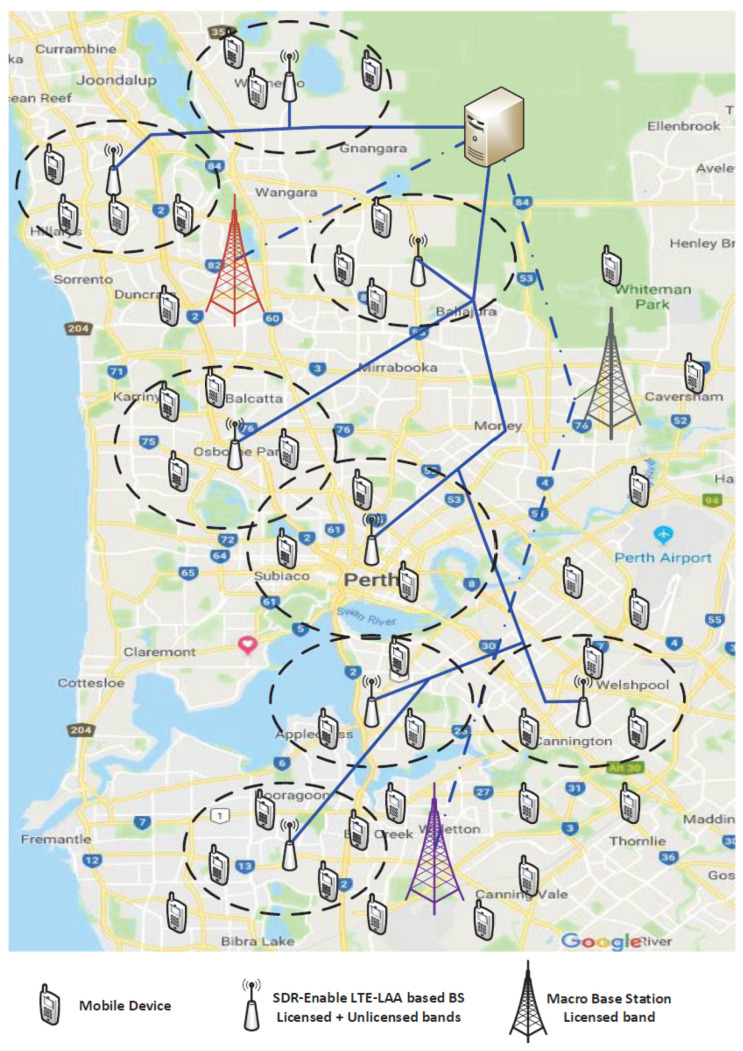
Hypothetical SDR-based system deployment.

Figure 3 displays a hypothetical deployment of the SDR-based system in the business district area of Perth, which is a major city in the state of Western Australia. In order to benchmark our proposed scheme, the system model shown in Figure 3 was implemented in MATLAB R2015b.

Table 1 lists the values of the used parameters in our simulations. In the simulation model, three macro base stations with a 1000 m radius [57] were implemented. All the SDR-based small-cell BSs were assumed to have circular coverage shapes and uniform distribution across the areas covered by the macro base stations. Additionally, subcarriers were randomly and uniformly distributed across the BSs. In our simulation, because the size of the users’ data followed a general distribution, the average data size was set to be 200 Kbits [24]. Furthermore, the arrival of users was assumed to follow the Poisson process.

**Figure 4:**
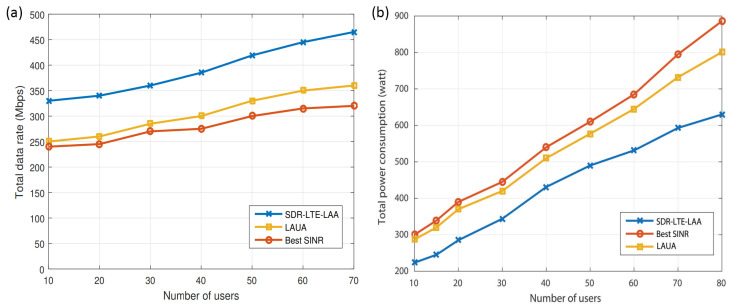
(**a**) Data rate and (**b**) power consumption comparison between LAUA, best SINR, and SDR-LTE-LAA approaches.

Figure 4a presents a comparison between our proposed approach (SDR-LTE-LAA), the best SINR, and the LAUA approach in terms of the total network data rate under a different number of users. Based on the results shown in Figure 4a, it is evident that our proposed SDR-LTE-LAA approach could deliver a considerably higher data rate than the other two approaches. Due to the utilization of LTE-LAA and SDR technology, our proposed approach tended to assign users continuously to the BSs which had more subcarriers and were closer to the users, irrespective of their operator, to provide high data rates and less communicating power consumption. However, the LAUA and B-SINR approaches tended to assign users to the BSs which belonged to the users’ operators and had lower loads or the best SINR, respectively. This happened irrespective of the distance between the user and the BS and resulted in some small-cell BSs becoming overwhelmed rather quickly, and fewer subcarriers per user could be achieved. Hence, our proposed approach performed better in terms of the total achieved data rate.

Figure 4b demonstrates a comparison between the total power consumption in our proposed approach and the other two approaches under a different number of users. Based on the results shown in Figure 4b, it is evident that the users consumed less power in our approach compared with the best SINR and LAUA approaches. This is because the other two approaches (best SINR and LAUA) assigned users only to their vendors’ BSs and based on the SINR or load on the BSs. Hence, they consumed more power to reach their vendors’ BSs and not to the closest BSs, as in our approach. From Figure 4a,b, it is clear that our proposed approach showed a tangible enhancement in terms of power savings and throughput in comparison with the best SINR and LAUA approaches. Furthermore, the SDR technology made the access network more flexible and helped the BSs to adapt to the transmission parameters according to the users’ locations and their required data rate. It is worth noting that in some cases, some base stations enter a situation where no users have been allocated to them. In such a situation, the sleeping mode concept can be utilized to reduce the overall network power consumption.

**Figure 5:**
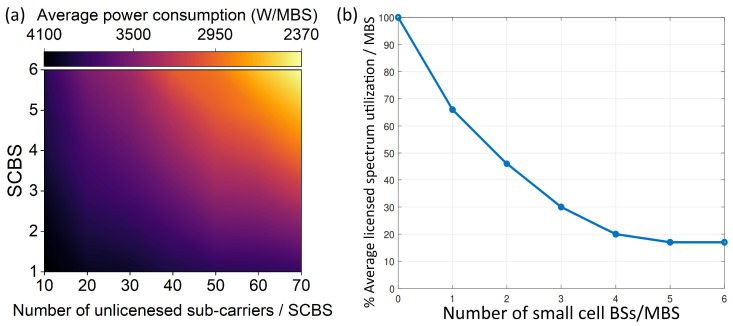
(**a**) Average power consumption for different SCBSs and number of carriers. (**b**) Average licensed spectrum utilization as a function of the number of small-cell BSs and MBSs.

In our work, we also investigated the influence of changing the number of SDR-based BSs and the amount of their available unlicensed bands on our proposed approach. Figure 5a presents the influence of changing the number of subcarriers in the unlicensed band on the average power consumption per macro base station. We studied the impact under a different number of small-cell BSs. From Figure 5a, it is clear that increasing the number of subcarriers resulted in a decrease in the macro BS’s average power consumption. This happened because when the number of subcarriers at small-cell BSs increased, more subcarriers became available at the SCBSs, and the system tended to offload users from macro base stations to the nearest SCBSs to achieve higher throughputs. This resulted in less traffic at the macro BSs, and consequently, the macro BSs consumed less power.

Figure 5b displays the influence of changing the number of small-cell BSs per macro BS on the utilization of the macro BSs’ licensed spectrum. From Figure 5b, it is clear that an increase in the number of small-cell BSs decreased the utilization of the macros’ licensed spectrum. This reduction occurred because the small-cell BSs supported the LTE-LAA architecture; hence, they utilized the unlicensed bands along with the licensed band to exchange data with nearby users, which alleviated the traffic through the licensed spectrum. Moreover, the proposed algorithm tended to assign users to small-cell BSs which had the highest subcarrier number in the unlicensed band. Accordingly, the users left macro BSs which utilized the licensed spectrum only to connect to small-cell BSs which had more carriers in the unlicensed spectrum or band. Subsequently, the percentage of the utilized licensed spectrum reduced. Based on our simulation results, it is evident that our proposed approach can decrease the licensed spectrum utilization per macro base station down to 20%. This reduction can be utilized in reallocating the unutilized bands or channels to other overloaded small-cell BSs to serve even more users or to other macro BSs, depending on the vendors’ operating conditions. SDR is an enabler technology for this step.

**Figure 6:**
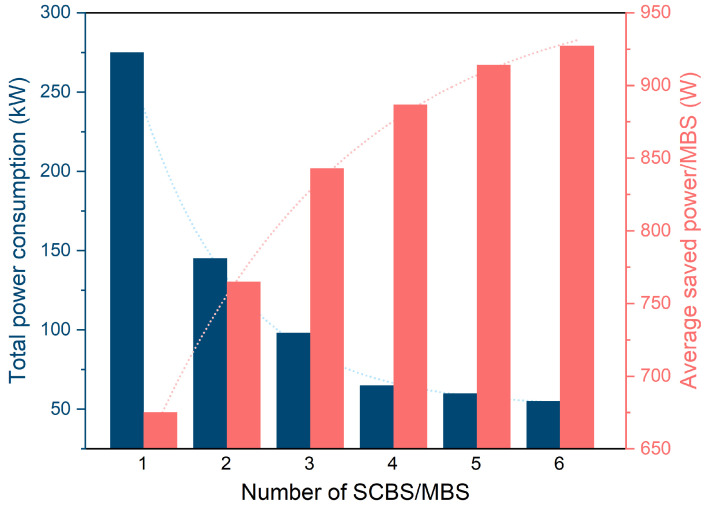
Average power saving per MBS and total network power consumption vs. number of SCBSs per MBS.

Figure 6 illustrates the influence of changing the small-cell BSs’ number per macro BS on the average amount of saved power per the vendors’ macro base stations. Based on the results in Figure 6, it is clear that increasing the small-cell BS number maximized the average amount of saved power per macro base station. This is because increasing the small-cell BS number caused an increase in the available number of unlicensed subcarriers, and this can be translated to higher achievable throughputs for the users. Accordingly, the algorithm showed a tendency to offload users toward the small-cell BSs, leaving the macro BSs. This offloading process resulted in a reduction in the traffic load at the macro base stations and, hence, their power consumption.

Figure 6 also presents the influence of changing the small-cell BS number on the overall network power consumption. From the results in Figure 6, it is evident that increasing the small-cell BS number decreased the overall power consumption. This is because increasing the small-cell BS number gives more options and opportunities for users, especially users at the edge of the macro cell, to offload the far macro base station and connect to any nearby small-cell BSs. Consequently, the proposed algorithm associates users with nearby small-cell BSs, leading to less power being consumed by the BS and users for communication. Additionally, the macro base stations with no users to serve are switched to sleep mode to save power. These factors are combined together to minimize the total network’s power consumption.

**Figure 7:**
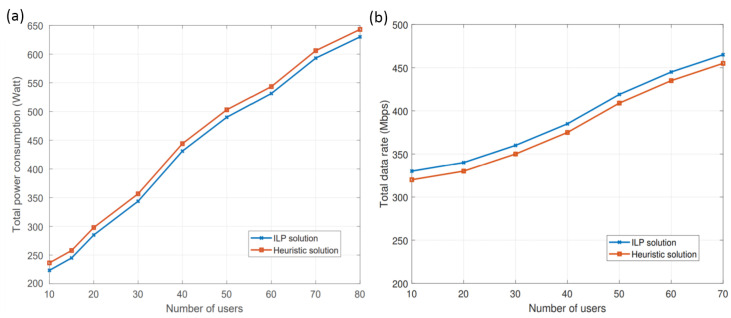
(**a**) Data rate and (**b**) power consumption comparison between the ILP results and our heuristic results.

Figure 7a,b shows comparisons between the results of the integer linear programming (ILP) solution and our heuristic solution. From Figure 7a, it is notable that our proposed heuristic results were marginally lower than those for ILP. Hence, our proposed heuristic can be utilized to obtain near-optimal performance. From Figure 7b, it is evident that our heuristic results were slightly higher than those for ILP. Thus, our proposed heuristic can be utilized to obtain near-optimal performance. Table 2 presents a summary of the findings of this work. Additionally, it compares our proposed approach with other approaches.

Based on the results in Table 2, it is evident that our proposed SDR-based and LTE-LAA-supported strategy consumed less power in comparison with the other strategies by up to 17%. Regarding the total data rate, and based on the results in Table 2, our proposed strategy provides users with higher data rates and throughputs compared with the other techniques. In our proposed system, the SDR-based BSs can allocate more carriers to users by using LTE-LAA technology, which utilizes the unlicensed spectrum along with the licensed spectrum. During the performance evaluation process of our system, we found that 80% of the BSs utilized the unlicensed spectrum to increase their supported data rates and serve more users. However, the other techniques rely only on the licensed spectrum to interact with their users, which limits the available carriers and constrains the data rates and the number of serviceable users.

## 5. AI–Based Multi-Objective System Optimization

**Figure 8:**
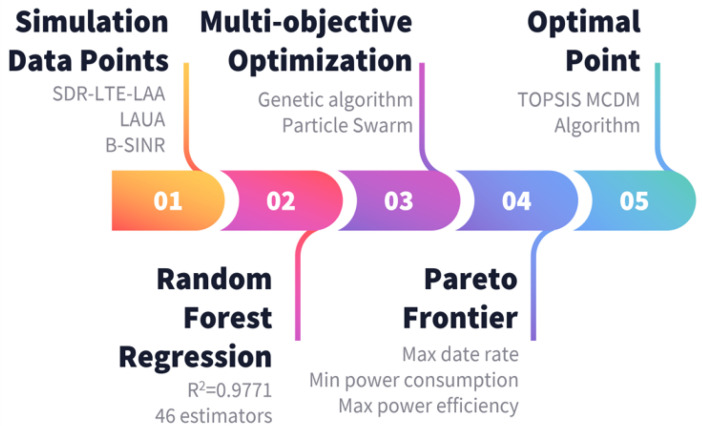
Flow chart of the complete AI-based multi-objective optimization process.

Next, we implemented two AI-based multi-objective optimization techniques, namely particle swarm optimization (PSO) and genetic algorithm (GA), in order to balance out the trade-offs between the attainable throughout and associated total power consumption. Figure 8 illustrates the entire optimization process, starting with obtaining the simulation results discussed in the previous sections.

**Figure 9:**
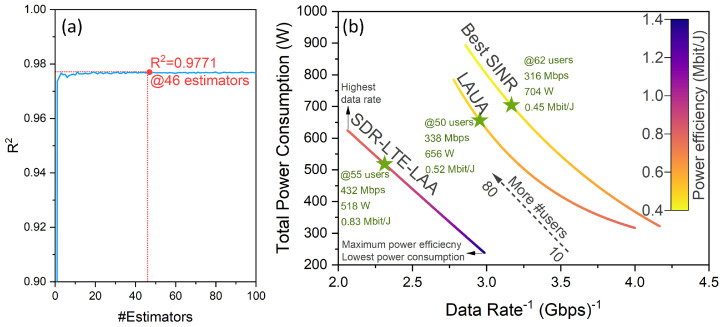
(**a**) Attained R-squared value based on the number of estimators used to train the random forest model. (**b**) The 3D design space, showing the three objective functions of the system depicting the Pareto frontier of each of the three approaches, alongside the TOPSIS optimal point, denoted by the green stars. Note the direction of the *X* axis. The power efficiency is color-coded at each point for each case. The dashed arrow indicates the direction of an increasing number of users from 10 to 80.

Initially, 80% of the obtained simulation results’ data points were used to train a random Forest Regression model, while the remaining 20% was used for validation purposes. In random forest regression, the R-squared parameter is a hyperparameter which indicates how well the model’s predictions match the actual data. As the number of estimators (trees) in the forest increases, the R-squared value typically improves because each additional tree helps to reduce variance and capture more of the underlying data structure. This leads to better model performance and a higher R^2^ value, which signifies a better fit. However, beyond a certain point, increasing the number of estimators (trees) can result in overfitting. That said, it was found that a number of estimators of 46 achieved the maximum prediction accuracy of 97.71%, as depicted in Figure 9a. Thereafter, the trained model was utilized as an optimization function in the multi-objective optimization investigation. We adopted three objective functions, namely maximizing the data rate, minimizing the power consumption, and hence maximizing the power efficiency (defined as the attainable data rate per Watt), all as functions of the number of system users.

In this part of the study, we used Python version 3.11.9 as the primary programming language due to its extensive libraries and ease of use for machine learning tasks. Specifically, we utilized the scikit-learn library, which provides a comprehensive suite of tools for data analysis and machine learning. The random forest regression model was implemented using the RandomForestRegressor class from scikit-learn. The DEAP library was utilized for the GA (Non-Dominated Sorting Genetic Algorithm III (NSGA-III)) and PSO for the optimization algorithms. The computational environment was configured on a system equipped with a 5.2 GHz-clocked Intel i9-10850K CPU with 64 GB of RAM. Table 3 shows the different hyperparameters adopted for the utilized algorithms of random forest regression, PSO, and NSGA-III, along with the rationale behind each hyperparameter selection.

With that said, both AI optimization techniques (i.e., GA and PSO) resulted in the same Pareto frontier consisting of a set of Pareto optimality points, defined as the set of points where no performance criterion can objectively be made better off. In other words, for each point outside of the Pareto frontier, there exists at least one point on the Pareto frontier which is objectively better in at least one performance criterion. Ultimately, the Pareto frontier serves as a guide to which points are viable to consider as a potential optimal point. As such, Figure 9b shows the obtained Pareto frontiers for both B-SINR and LAUA alongside our proposed SDR-LTE-LAA approach, in terms of total power consumption and data rate objective functions, for numbers of users between 10 and 80. It is worth mentioning here that the inverse of the data rate was utilized here, since our objective was to maximize it rather than minimize it. Furthermore, the associated power efficiency at each point along the Pareto curve is color-coded according to the depicted colormap. At first glance, Figure 9b clearly showcases the drastic advantages introduced by the SDR-LTE-LAA approach. Not only did it yield the highest attainable data rate and lowest power consumption, but it also achieved a significantly higher power efficiency (almost twofold higher). This is evident by following any horizontal line across the three curves indicating the attainable data rate for any given power consumption level. In other words, our SDR-LTE-LAA approach provides yet another vital feature, which is efficient green scalability, as the data rate and expected number of users grew per the requirements and expectations of next-generation communication systems.

Thereafter, to pick the optimal point for each of the three approaches, we implemented the Technique for Order of Preference by Similarity to Ideal Solution (TOPSIS), a multi-criteria decision-making (MCDM) algorithm developed by Hwang and Yoon [60,61] where each point is ranked according to its similarity to the ideal solution. The TOPSIS optimal point for each approach is indicated by the green stars in Figure 9b, which for the SDR-LTE-LAA approach was shown to have 55 users with a total power consumption of 518 W and data rate of 432 Mbps, resulting in a power efficiency of 0.83 Mbit/J. One the other hand, the corresponding values for the LAUA (B-SINR) approach were 656 (704) W, 338 (316) Mbps, and 0.52 (0.45) Mbit/J, realized with 50 (62) users, respectively. Evidently, our SDR-LTE-LAA approach showcased superior performance metrics across the board, with a 100 Mbps higher data rate compared with the other approaches and 150–185 W (20–26%) less power consumption for the respective TOPSIS optimal point for each case.

## 6. Conclusions

In this work, we introduced a flexible software-defined radio-based HetNet architecture which can promptly adjust to changing network requirements. The proposed framework combines LTE-LAA with SDR technology to optimize the network throughput while minimizing the overall power consumption within HetNets. We approached the resource management challenge by framing it as a constrained optimization problem, and our solution significantly outperformed existing methods. Our approach boasts a remarkable 17% reduction in energy consumption compared with current practices. Furthermore, in terms of the network data rate and utilization of the licensed spectrum, our solution offers an impressive 31% increase in data rate and nearly 22% utilization of the licensed spectrum courtesy of LTE-LAA technology adoption. We proved that the optimization problem was an NP-hard problem. For an NP-hard problem, time complexity may become prohibitively high when the number of parameters becomes high in the model. Considering the time complexity of solving an NP-hard problem and the desire to obtain a real-time solution, we also introduced a throughput- and energy-aware heuristic solution. The proposed heuristic was found to perform considerably better than other approaches.

In this study, we adopted random forest regression, PSO, and GA, due to their robustness, ability to handle large datasets with high dimensionality, and effectiveness in capturing complex, nonlinear relationships within the data (random forest regression). Furthermore, random forest regression’s ensemble nature helps reduce overfitting and provides a reliable performance benchmark for our analysis. This was demonstrated by the high agreement between the model’s prediction and the actual data, as evident by the 97% R-squared value. However, future work could explore alternative optimization approaches to further validate and potentially enhance our findings. Dynamic programming (DP) could be investigated for its strength in solving problems with an ideal substructure, despite its computational limitations in high-dimensional spaces. Additionally, deep reinforcement learning (DRL) offers a promising adaptive and learning-based framework which may provide novel insights and performance improvements, though it requires extensive training data and computational resources. Future research may consider these methodologies to compare their efficacy and robustness against the adopted methodology used in this study. Future research efforts could explore potentially incorporating network traffic analysis, latency measurements, and scalability considerations in a cross-layer architecture. In addition, the effect of the SDN controller placement (typically centralized for accessibility to all base stations in the network to track their traffic) can be investigated in detail as its own optimization parameter among other HetNet variables.

## Figures and Tables

**Table 1:** Parameters used in simulation [33,58,59].

Parameter’s Name	Value
Frequency of the Carrier	1.9 GHz
Bandwidth of the Channel	11 MHz
Number of Licensed Subcarriers	64
Bandwidth of Each Subcarrier	78 KHz
Capacity of the Traffic Model	Full Buffer
Macro BS Power Consumption	20 W
SCBS Power Consumption	5 W
Total Number of MBs	3
Total Number of SCBSs	7
Overall Coverage Area	5 km × 5 km
Path Loss Model	3GPP TR 36.814
Allocation of the Spectrum	Partitioned
Transmitter Gain	1 dB
Receiver Sensitivity	−97 dBm
Noise Power Level	−104 dBm
Antenna Pattern	Omnidirectional
Antenna Feeder Loss	3 dB

**Table 2:** Summary of the findings.

User–BS Association Strategy	Total Power Consumption (W)	Total Data Rate (Mbps)	Unlicensed Spectrum Utilization
Best SINR [55]	600	320	N/A
LAUA [56]	560	350	N/A
Proposed SDR-LTE-LAA	495	440	78

**Table 3:** Adopted hyperparameters for each of the utilized AI algorithms, along with the rationale behind the selection of each of them.

Algorithm	Hyperparameter	Value	Rationale
Random Forest Regression	Number of trees	46	Results in the maximum R-squared value
	Maximum depth	None	Allows trees to expand fully to capture more complex patterns
	Minimum samples to split	2	Default value to ensure all potential splits are considered
	Criterion	MSE	Mean squared error (MSE) is used as a standard measure for regression problems
NSGA-III	Population size	100	Provides a diverse set of solutions to explore the search space effectively
	Crossover rate	0.9	To encourage more genetic mixing, which is beneficial for diversity
	Mutation rate	0.01	To maintain diversity and avoid premature convergence
	Number of generations	50	To ensure sufficient iterations for the population to evolve and converge toward optimal Pareto fronts
	Selection method	Crowded tournament selection	To ensure diversity among solutions by considering both rank and crowding distance
PSO	Swarm size	50	Provides a sufficient number of particles to explore the search space effectively
	Social coefficient	2.0	Encourages particles to move toward the best positions found by the swarm
	Cognitive coefficient	2.0	Ensures particles are influenced by their own best-known positions
	Inertia weight	0.7	Balances exploration and exploitation in the search process

## Data Availability

Data are contained within the article.

## Abbreviations

The following abbreviations are used in this manuscript:

VNI	Visual networking index
HeNets	Heterogeneous networks
BS	Base station
SCBS	Small cell base station
SDR	Software-defined radio
LTE-LAA	Long-term evolution licensed assisted access
3GPP	3rd Generation Partnership Project
OFDMA-PON	Orthogonal frequency division multiple access
SNIR	Signal-to-interference-and-noise ratio
HCA	Hybrid channel assignment
LBSB	Load balancing with selective borrowing
CBWL	Channel borrowing without locking
MBS	Macro base station
MCS	Modulation and coding scheme
PP	Poisson process
TEA	Throughput- and energy-aware
LTE-LAA	Long-term evolution licensed assisted access
TOPSIS	Technique for Order of Preference by Similarity to Ideal Solution
MCDM	Multi-criteria decision making
GA	Genetic algorithm
PSO	Particle swarm optimization
LAUA	Load-aware user association
B-SINR	Best signal-to-interference-and-noise ratio
DP	Dynamic programming
DRL	Deep reinforcement

Table of Notations
The following notations are used in this manuscript:

*J*	User number
*I*	BS number
$L_{1}$	Unused or idle bands
$L_{2}$	Nearby macro-connected users and their utilized channel frequencies
$L_{3}$	Optimum user–BS association list
$P_{i}$	BS transmission power
$g_{ij}$	Gain of the channel between a BS *i* and a user *j*
$\sigma^{2}$	Noise power
$\alpha_{ij}$	SINR from a BS *i* toward a user *j*
γ	Channel attenuation factor
$P_{e}$	Symbol error probability
*m*	Modulation order
$P_{e}^{b}$	Bit error probability
$P_{ef}^{blk}$	Probability to receive an error-free block
*W*	Codeword width
*Q*	Number of bits
$P_{e}^{blk}$	Block error probability
$D_{ij}^{max}$	Max data rate between the BS *i* and user *j*
$N_{i}^{c}$	Total number of assignable subcarriers at BS *i*
$N_{l}^{i}$	Number of subcarriers in the licensed band
$N_{u}^{i}$	Number of subcarriers in the unlicensed band
$N_{symb}$	Number of symbols
$R_{w}$	Code rate of the used MCS
$T_{fr}$	Frame time duration
$l_{j}$	Poisson average value
$r_{j}$	Poisson arrival rate
$\epsilon_{ij}$	Average traffic load density in BS *i*
$x_{ij}$	Binary variable showing whether *j*th user is connected to the *i*th BS
$\varphi_{i}$	Average traffic load density for a BS *i*
$T_{ij}$	Average time of service for user *j*
$D_{ij}^{eff}$	Throughput for each user *j* connected to BS *i*
$\eta_{i}$	Total throughput of a BS *i*
pj	User *j*’s total power consumption
$pj^{dy}$	User *j*’s dynamic power consumption
$pj^{st}$	User *j*’s static power consumption
ψj	Coefficient representing the relationship of data traffic load and dynamic power
Pij	User *j*’s transmission power
*C*	Speed of light in vacuum
dij	Distance between user *j* and BS *i*
*G*	Antenna gain
$f_{z}^{i}$	Frequency of the assigned subcarrier *z* of BS *i*
$p_{j_{max}}$	Frequency of the assigned subcarrier *z* of BS *i*
β	Trade-off coefficient
